# Biological Evaluation of Zinc Phosphate Cement for Potential Bone Contact Applications

**DOI:** 10.3390/biomedicines11020250

**Published:** 2023-01-18

**Authors:** Arun K. Kotha, John W. Nicholson, Samantha E. Booth

**Affiliations:** 1 Department of Pharmaceutical Sciences, College of Pharmacy, Mercer University, Atlanta, GA 30341, USA; 2 Bluefield Centre for Biomaterials, 152-160 City Road, London EC1V 2NX, UK; 3 United Kingdom and Dental Physical Sciences, Barts & The London School of Medicine and Dentistry, Queen Mary University of London, Mile End Road, London E1 4NS, UK; 4 School of Science, University of Greenwich, Medway Campus, Chatham Maritime, Kent ME4 4TB, UK

**Keywords:** zinc phosphate, cement, MTT assay, SEM, anti-microbial

## Abstract

Zinc phosphate cement is used in dentistry to lute crowns and bridges. So far, its biocompatibility for other applications has not been studied. This paper reports the biocompatibility of zinc phosphate towards MG63 cells, testing both the material (discs; 3 mm diameter × 1 mm thick) and leachate from the cement. Cell viability was determined using an MTT assay, and cytotoxicity from the effects of leachate, studied in triplicate. Microscopy (optical and scanning electron) determined the morphology and proliferation of cells attached to zinc phosphate. ICP-OES measured element release into leachate, and anti-microbial behaviour was determined against *Streptococcus pyrogenes* cultured on a Brain Heart Infusion agar using cement discs (3 mm diameter × 1 mm thick). Zones of inhibition were measured after 72 h. MG63 cells proliferated on zinc phosphate surfaces and retained their morphology. The cells were healthy and viable as shown by an MTT assay, both on cement and in leachate. High levels of phosphorus but low levels of zinc were released into leachate. The cement showed minimal antimicrobial activity against *S. pyogenes*, probably due to the long maturation times used. Zinc phosphate cement was found to be biocompatible towards MG63 cells, which indicates that it may be capable of use in bone contact applications.

## 1. Introduction

Zinc phosphate cement has been used in dentistry for more than a century. It is used for various clinical applications, such as luting crowns and bridges [1,2] and cementing onlays [2]. It belongs to the acid–base cement group [3] and its acidic component consists of a solution of phosphoric acid (45–65%), which also contains aluminium (1–3.1%) and zinc (up to 10%) [2]. Aluminium and zinc play a critical role in controlling the rate of reaction, which they do by forming appropriate amounts of phosphates in a solution, and these increase the pH of the acid solution and reduce its reactivity. Set cements contain water in some sort of chemical combination, there being no phase-separation as the cement sets. The chemical and mechanical properties of the fully reacted cement are critically dependent on the concentration of the phosphoric acid in the initial solution [4], and for this reason the liquid component must not be allowed to gain or lose water to the atmosphere [5].

The base component is usually a powder containing mainly zinc oxide (at least 90%) and also magnesium oxide (3 to 10%). The MgO is used as modifier to reduce the reactivity of the zinc oxide towards the phosphoric acid solution. Reactivity is also controlled by sintering the zinc oxide at temperatures from 1000 to 1350 °C. This also drives off a very small amount of oxygen and causes the oxide to become very slightly non-stoichiometric, with a formula corresponding to ZnO_(1−x)_ (x up to 70 ppm) [6,7]. The resulting material is slightly yellow in colour and also less reactive towards acids. 

The reaction between zinc oxide powder and aqueous phosphoric acid is strongly exothermic, even in their deactivated forms. Because of this, it is necessary to mix the components on a cool slab, over a wide area while incorporating small increments of powder into the liquid for approximately 1.5 min. After mixing, the pH of the cement is approximately 3.5, a value that can lead to pulpal irritation and post-cementation sensitivity in patients [1]. After 24 h, when fully reacted, the set cement reaches a pH of 6.7 [2], a value which is much more acceptable biologically. Studies have shown that zinc phosphate dental cement is moderately cytotoxic, and has greater damaging effects when freshly mixed than when fully set [8,9]. However, it has an excellent record in clinical use and is preferred by many clinicians, despite the fact that other types of luting cement are available [9,10].

Zinc phosphate is considered biocompatible when used in dentistry, with reports of it being used without long-term adverse effects for times exceeding 40 years [10]. Consequently, it may also have the potential for use as a biomaterial in contact with bone. There are a number of pieces of evidence to suggest that this possibility is worth considering. First, zinc is known to be an essential trace element that, in the form of Zn^2+^ ions, stimulates bone formation [11,12]. Second, adding zinc to phosphate glasses has been found to be beneficial when those glasses are used for orthopaedic tissue engineering [13]. Third, coating metallic zinc implants with zinc phosphate has been found to improve bone-contact biocompatibility of those implants [14].

These findings suggest that studying the biological behavior of zinc phosphate dental is of interest in order to assess its potential for use in bone contact applications. A widely used artificial material in this application is poly(methyl methacrylate) [15], used for the fixation of metallic and polymeric prostheses in both hip- and knee-replacement surgery. In recent years, there has been a move away from the use of such bulky artificial materials and increasing interest in tissue-engineered bone developed on synthetic scaffold materials [16,17,18,19,20]. A variety of materials have been studied for this, including composites of various types based on polymers such as collagen and poly-L-lactic acid [17], carboxymethyl cellulose [18,19] and polycaprolactone [19]. There has also been work on speciality glasses to promote bone growth [16]. These studies have typically evaluated the biological properties of the new materials using cell culture, typically with MG63 cells, though other cell types have also been used.

Zinc phosphate cement may have the potential for a wider application on bone contact devices, depending on its interactions with living cells. It is known to have a setting exotherm, though one that is lower than that of poly(methyl methacrylate). Unlike some of the systems studied, zinc phosphate will not release free monomer, because it does not contain any. However, so far this cement does not seem to have been considered for use in bone-contact applications. The current paper aims to address this issue, and in particular to consider the biological behavior in an appropriate cell culture. The hypothesis on which this study is based is that zinc phosphate has acceptable biocompatibility against cultured cells, thereby demonstrating its suitability for further investigation as a material to be used in contact with bone. 

## 2. Materials and Methods

### 2.1. Cell Culture

Zinc phosphate cement samples (DEHP, Kent Express, Gillingham, UK) were prepared by mixing small amounts of modified zinc oxide powder into deactivated phosphoric acid solution using a spatula on a cooled glass block, as recommended by the manufacturer. Freshly mixed cement pastes were transferred to specially made acrylic moulds to prepare the discs of size 3 mm diameter × 1 mm thick. Cement samples were sterilised by applying UV light for 3 h on each side of the sample. Each sample was then incubated with 1 mL of complete cell culture media for 24 h at 37 °C to create a leachate.

MG63 osteosarcoma cells were grown as a monolayer culture in 75 cm^2^ cantered neck flasks, as has previously been described in the literature [17]. Cells were supplemented with cell culture media containing DMEM, 10% heat inactivated bovine serum and penicillin (100 units/mL), streptomycin (100 µg/mL) and glutamine (0.292 mg/mL). They were passaged once every 4 days and were maintained at 37 °C and 5% (*v*/*v*) CO_2_.

Adherent cells at lag phase were detached by applying 1 mL of TrypLE^TM^ Select and incubated at 37 °C for 5–10 min. Once they were detached from the substrate, cells were suspended in 9 mL of complete cell culture media. Cells were counted using a haemocytometer and were seeded into 24 well plates at a density of 1 × 10^4^ cells/mL (2 mL cell suspension per well). After 24 h of incubation at 37 °C, cell culture media was removed and 0.5 mL of fresh media was added to the cells together with 0.5 mL of zinc phosphate leachate and incubated at standard incubation conditions. Cell viability was measured using MTT (3-(4,5-dimethylthiazol-2-yl)-2,5-diphenyltetrazolium bromide) [20,21]. The assay was performed at three different time points (24, 72 and 120 h). Non-treated cells were used as controls [22].

MTT reagent was prepared at 0.5 mg/mL concentration in phosphate buffered saline (PBS, pH 7.4) and sterilised by passing through 0.22 µm filter. After the incubation times, 200 µL of MTT reagent was added to all wells and incubated for 4 h at standard incubation conditions. After the incubation period, the media was discarded from the wells and 2 mL of DMSO was added and incubated at standard incubation conditions for 30 min. From each well, 200 µL of solution was transferred into a 96 well plate, and plates were then read at 540 nm using a microtiter plate reader. The results were expressed as cell viability (%) (±standard error of the mean) against incubation times.

The cytotoxicity tests were performed in triplicate on the zinc phosphate cement leachate. During the MTT assay, absorbance of viable cells was converted into a percentage, assuming the control (untreated) cell absorbance is equal to 100% viability. Polyethylene imine, PEI, was used as a positive control, due to its cytotoxicity.

### 2.2. Optical Microscopy

The osteosarcoma cells were seeded at a density of 1 × 10^4^ cells/mL (2 mL cell suspension per well) into 24 well plate. After 24 h, the media was discarded and 500 µL of fresh cell culture media was applied together with 500 µL of leachate. Polyethylamine, with final concentration of 100 µg/mL, was used as positive control and non-treated cells were used as a negative control. After 72 h of incubation, images of cells were taken using an inverted microscope.

### 2.3. Scanning Electron Microscopy (SEM)

Images were taken at four different time points (3, 5, 7 and 14 days) on cement discs prepared as previously described. The samples were individually adhered onto glass cover slips (10 mm) using conductive carbon cement and sterilised by exposing the disc to UV light for 3 h on each side. Each sample was placed into a well on a 24 well plate. MG63 cells were seeded at a density of 1 × 10^4^ cells/well (2 mL) into wells. PEI was applied as a positive control and un-treated cells were used as negative controls. After 3 days of incubation, one well plate was removed from the incubator. Media was discarded from the wells and the cells were fixed by using 1 mL of 2.5% glutaraldehyde and stored for 1 h at room temperature. After 1 h, glutaraldehyde was discarded and cells were washed with 2 mL PBS twice. After the cells were washed, 2 mL of 1% osmium tetroxide was applied into each well and incubated at room temperature for 1 h. After 1 h, cells were washed with PBS as mentioned previously. After the PBS washings, cells were dried using increasing concentrations of graded ethanol from 30, 40, 50, 70, 80, 90, 95 and 100%. Each concentration was applied and stored for 20 min. This process was repeated once more. Finally, the cells were dried completely by pipetting 2 mL of hexamethyldisilazane (HMDS) into each well and storing for 20 min. Once dry, samples were coated with chromium (Emitech K575X Sputter Coater) before SEM analysis. All images were taken 1000× magnification.

### 2.4. ICP-OES

After seeding osteosarcoma cells (2 × 10^4^/well) in a 24 well plate, they were incubated for 24 h at standard conditions (37 °C, 5% *v/v* CO_2_). A 3 mm diameter × 1 mm thick disc of zinc phosphate cement prepared as previously described and sterilised (3 h each side with UV light) added to the seeded wells. After 48 h, the leachate was collected and digested with concentrated nitric acid for two hours. Then, 1 mL of this concentrated solution was diluted by adding 9 mL of distilled water and placing in a centrifuge tube ready for analysis. The leachate of seeded wells with no zinc phosphate cement was used as a blank to ascertain which elements were leached out of the cement into the media. The same digestion method was used as above. The diluted samples were analysed for zinc and phosphorus using an Optimal 4300 DV instrument (PerkinElmer, Waltham, MA, USA). The results are shown in mg/L. 

### 2.5. Antimicrobial Studies

*Streptococcus progenes* was cultivated in 10 mL of Brain Heart Infusion broth for 24 h at 37 °C. From this stock bacterial culture, 100 µL of bacterial suspension was spread onto the Brain Heart Infusion agar plates and incubated for 24 h at 37 °C. 

A single colony of bacteria was transferred into 10 mL of BHI broth and incubated overnight at standard incubation conditions. The single colony culture was used for the antimicrobial test. The culture was centrifuged at 4000 rpm for 10 min (Accuspin 1 centrifuge, Fisher Scientific, Warrington, UK). The supernatent was discarded and 10 mL of sterile PBS was added and centrifuged at 4000 rpm for 10 min. The supernatent was again discarded and the bacterial pellet was suspended in 10 mL of sterile PBS, which was used as the stock solution in further analysis.

The stock solution of bacterial cells was diluted at 1/2, 1/4, 1/8, 1/16 and 1/32 ratios and the absorbance was measured at 500 nm using spectrophotometer. An additional set of dilutions were made (10^−2^, 10^−4^, 10^−6^ and 10^−8^). From the 10^−6^, 10^−8^ stocks, 100 µL and 1 mL were transferred on to fresh BHI agar plates and incubated at standard incubation conditions for 24 h. A cell count was performed for these four plates. Finally, a bacterial cell count in the stock solution was measured using the absorbance values and cell counts from BHI agar plates.

In a fresh BHI agar plate, *Streptococcus pyogenes* was seeded at 5 × 10^5^ CFU/mL and a disc (3 mm × 1 mm) of freshly made zinc phosphate cement was placed in the centre of the plate. Penicillin (10 units) was used as a positive control on a separate plate and both plates were incubated at standard conditions for 72 h. After the time period had elapsed, the zone of inhibition was measured. 

### 2.6. Statistical Analysis

Multiple independent experiments were performed and results were expressed as means and standard deviations. Among groups, differences were tested for significance using one-way ANOVA followed by Tukey’s HSD test using GraphPad PRISM, Version 5a (San Diego, CA, USA). Differences of at least *p* < 0.05 were considered significant.

## 3. Results

### 3.1. Biocompatibility (Cytotoxicity) Studies

**Figure 1:**
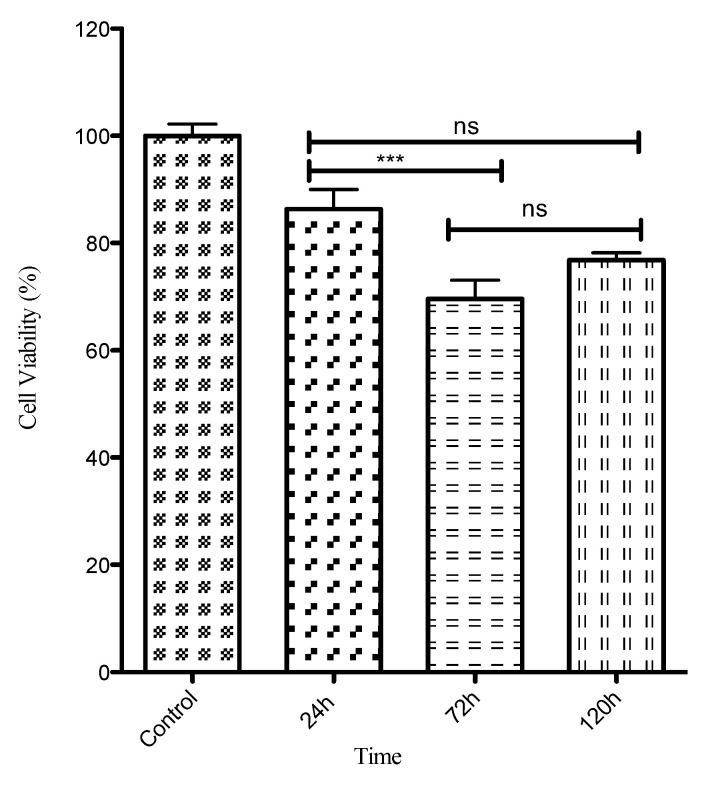
MTT cell viability assay of zinc phosphate leachate on MG63 cells after incubation periods of 24, 72 and 120 h compared with control group (untreated cells), shown as 100% (*** significant to *p* < 0.05).

Figure 1 shows that MG63 cells remain viable when contacted with zinc phosphate cement leachate at three different time points. They were found to remain viable; after 24 h, cell viability was 86%. After 72 h, cell viability was more than 71% and, interestingly, after 120 h, cell viability had increased to 76%. The differences between the result at 24 h and those at both 72 h and 120 h were statistically significant (*p* < 0.05). However, these results indicate a high level of cell viability, even at 120 h, suggesting that zinc phosphate cement is biocompatible with MG63 cells. The mitochondrial dehydrogenases in the cells did not lose the capacity to reduce the MTT reagent, which was evident from the high cell viability.

### 3.2. Microscopy

**Figure 2:**
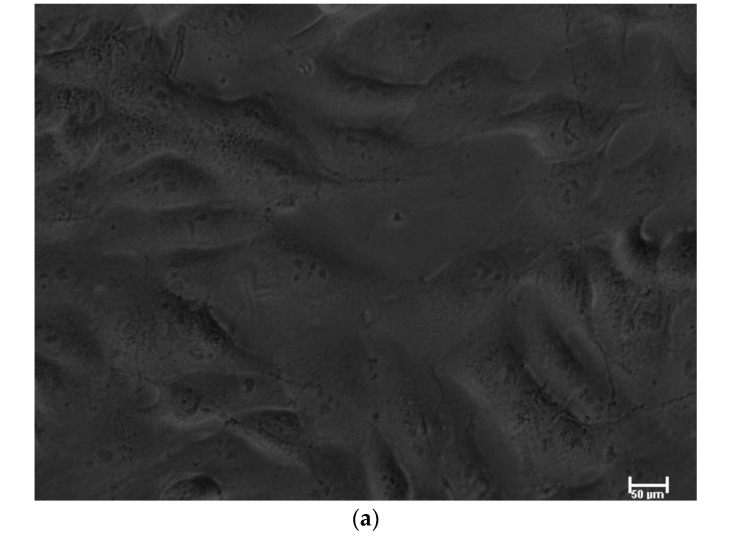

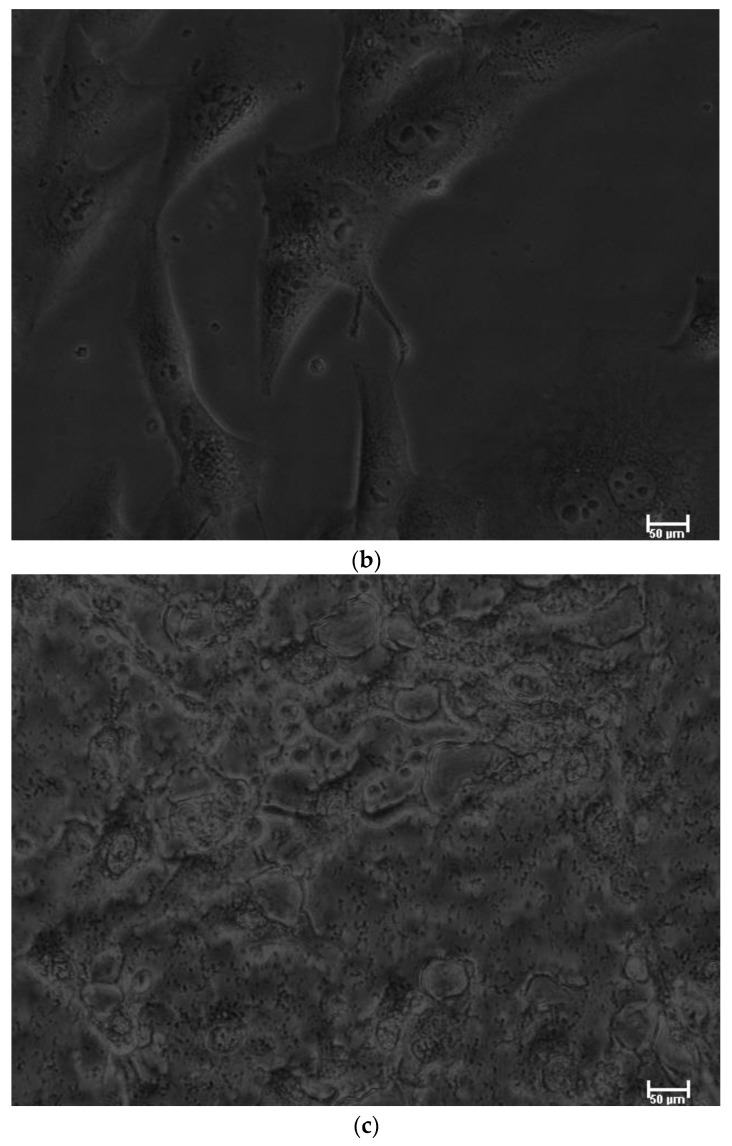
(**a**): MG63 control, cells not in contact with leachate. (**b**): MG63 cells in contact with leachate from zinc phosphate cement, 72 h, showing cells are viable. (**c**): MG63 cells in contact with PEI for 72 h, showing complete destruction of cells.

Optical microscopy showed that the MG63 cells in contact with zinc phosphate leachate had no major changes compared with the control cells (Figure 2a–c). By contrast, PEI completely destroyed the cell morphology. These results confirm those of the cytotoxicity experiments, which showed that viability remained above 70% after 72 h and 120 h of incubation with the zinc phosphate cement.

### 3.3. SEM

**Figure 3:**
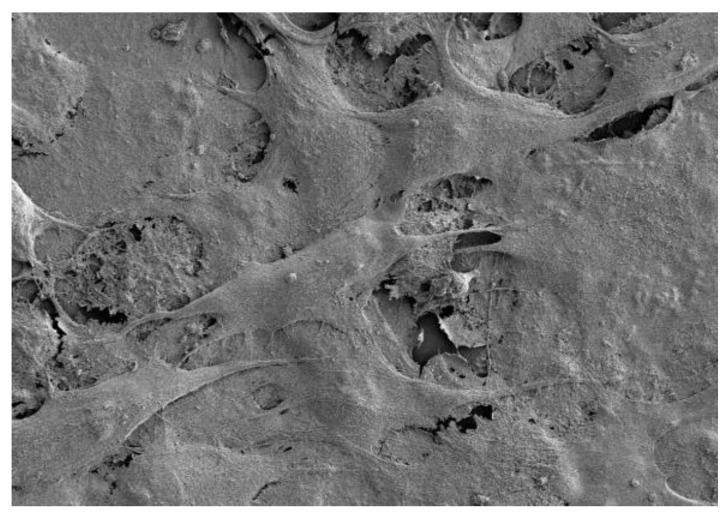
MG63 cells on zinc phosphate after 14 days.

**Figure 4:**
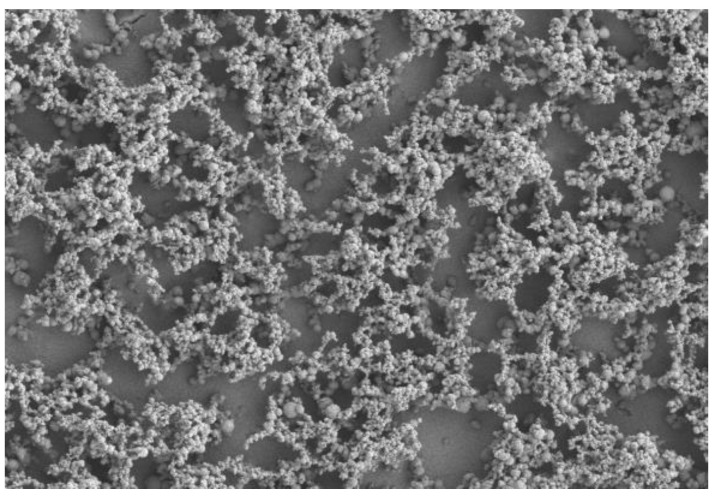
Cells exposed to PEI, showing extent of destruction after 3 days.

The cells in Figure 3 show the complete coverage of the surface after 14 days with a horizontal stacking pattern. The polyethylene imine, PEI, sample in Figure 4 shows the complete destruction of cell morphology due to cytotoxicty. SEM was able to show that, after 3 days of incubation, healthy osteoblast cells were communicating on the crystalline surface of the zinc phosphate cement. After 5 days, the cells started to embed and adhere to the surface, and after 7 days, the cells show distinct signs of growth. By 14 days, the MG63 cells had become dense and formed layers.

### 3.4. ICP-OES

**Figure 5:**
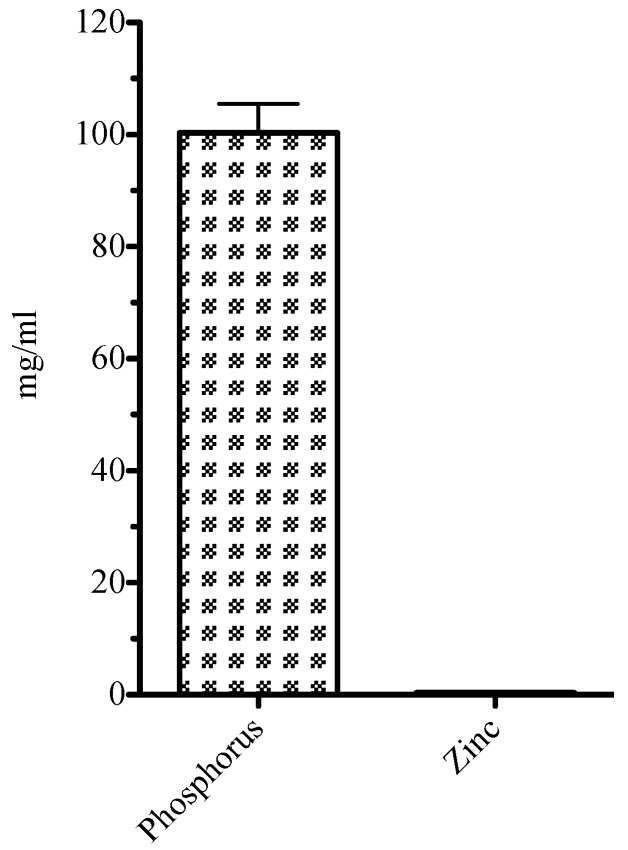
Phosphorus and zinc release from cement.

Figure 5 shows ion release into cell culture media after 48 h incubation. The zinc release was minimal at 0.322 mg/L, whereas the phosphorus release showed 100.337 mg/L in the cell culture media. 

### 3.5. Antimicrobial Studies

**Figure 6:**
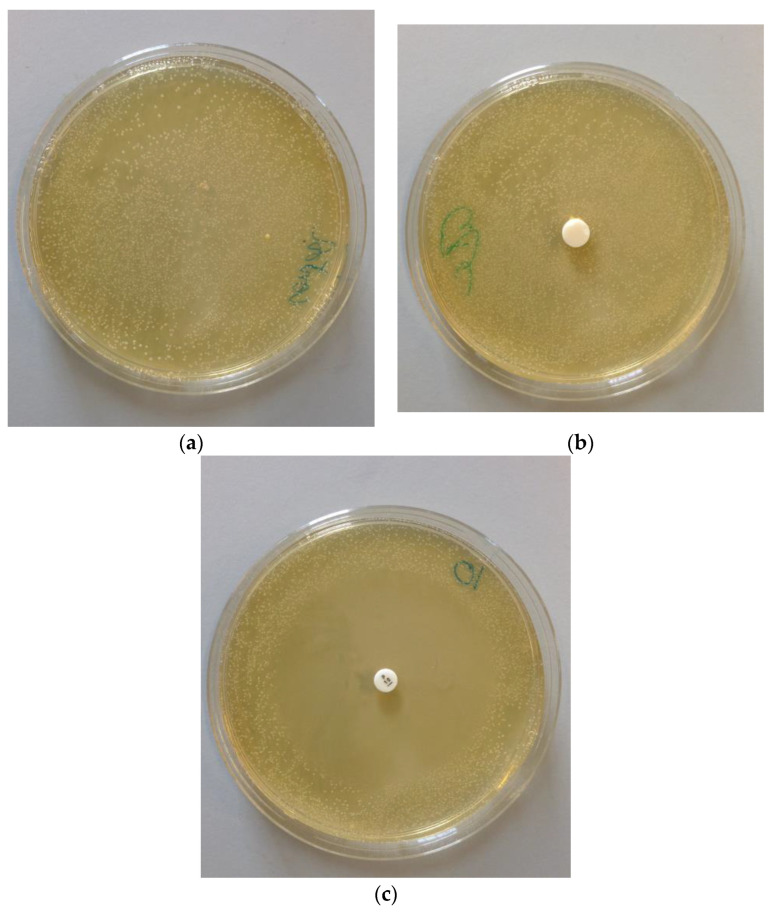
(**a**): *S. pyogenes* after 48 h. (**b**): Zinc phosphate speciment in *S. pyogenes*. Note the extensive bacterial growth. (**c**): Penicillin as positive control, showing zone of inhibition.

Figure 6a shows *Streptococcus auries* bacteria after 48 h. This was used as a control for the antimicrobial study and shows a layer of bacterial colonies on the surface of the plate. Figure 6b shows zinc phosphate cement incubated for 48 h and exhibiting similar bacterial growth to the control. Figure 6c shows penicillin as a positive providing a clearly visible zone of inhibition around the sample where there are no *S. auries* bacteria.

## 4. Discussion

The results obtained enable us to draw clear conclusions concerning the biocompatibility of zinc phosphate towards MG63 osteosarcoma cells and the micro-organism *S. pyogenes*.

MG63 cells were chosen because they are a distinct cell line and genetically identical to each other. This means that experiments with them are standardized and, in principle, reproducible [22,23,24]. Although they are osteosarcoma cells, they possess a number of the features of osteoblasts [25] and behave similarly in terms of colonisation of surfaces and proliferation [26]. Because of this, they have been widely used in biocompatibility studies on a range of materials under consideration for use in bone-contact applications. These include novel titanium alloys [26], mineral trioxide aggregate [23] and phosphate glasses [24].

*S. pyogenes* was used as a general-purpose micro-organism because it is widely available and convenient to use [27]. It typically infects the skin or oropharynx of humans, with mild results. However, it can invade the body, leading to sepsis, with results that may be life-threatening.

In the present study, MG63 osteosarcoma cells were observed to attach, spread and proliferate when cultured onto the surface of the zinc phosphate cement. This shows that the cement is highly biocompatible towards these cells and indicates that the zinc phosphate cement is suitable for use in bone-contact applications.

This high level of biocompatibility was confirmed with the leachate from the zinc phosphate cement, where the MTT assay demonstrated 76.8% viability of MG63 cells over 120 h when compared with the control cells. This result confirms that zinc phosphate cement has the potential for use as a bone contact material. Although this was the only test of cell viability used, its use as the sole test of viability is typical in studies of this type [28,29]. This test is the one recommended in the appropriate ISO Standard for the biological evaluation of materials [30], due to its high reliability [31].

ICP-OES analysis of the leachate showed that both zinc and phosphorus was released from the cement, with phosphorus being released in much greater amounts. This contrasts with previous findings of ion leaching from this type of cement, where three different extraction media were used (deionised water, lactic acid solution and lactate buffer solution) [4]. However, in this study all three media had acidic pH values, even the water, which was pH 5.9. The present extraction media were designed to mimic the pH of body fluids such as serum and these are typically very slightly alkaline. Previous studies have shown that most of the zinc is released from unreacted ZnO within the cement, and hence arises due to an attack by the acidic storage medium on the basic filler [4]. Storing cements in mildly alkaline media suppresses this zinc release and favours phosphorus release, almost certainly in the form of phosphate ions, PO_4_^3−^.

The morphology of the osteosarcoma cells was studied using an inverted optical microscope and also scanning electron microscopy, SEM. From both microscopy techniques, it was clear that after 48 h contact, the morphology of the MG63 cells did not alter in comparison with the control cells. These micrographs are consistent with the toxicity data, and show that over this early timescale, the zinc phosphate cement is highly biocompatible towards these cells.

With longer time intervals, the MG63 cells proliferated on the surface of the cement. As they did so, their morphology changed and, by 14 days, they occupied most of the surface. They also grew on top of each other into layers, and eventually produced a well-integrated colony over the entire zinc phosphate surface. In this way, the high long-term biocompatibility of the zinc phosphate cement to MG63 cells was demonstrated. These results confirm that the cement has the potential to be used in applications where direct contact with bone is needed, such as in the repair of bone defects and as a scaffold for bone growth.

Currently, self-hardening calcium phosphate cements are used in this way [32], but they have a low mechanical strength and this limits their clinical uses. The typical values of the compressive strength of calcium phosphate lie between 30 and 65 MPa, depending on composition and also on whether they have been reinforced [32,33,34]. These cements have excellent biocompatibility towards bone [32] and have a number of applications that involve placing them in direct contact with human trabecular bone. Our findings suggest that zinc phosphate cement could also be used in this way, because of its good biocompatibility towards osteoblast-type cells. It also has the advantage of higher compressive strengths, which are typically over 100 MPa, and possibly up to 130 MPa [34].

Lastly, the anti-microbial properties of zinc phosphate cement are also determined in the current study. Experiments used specimens that had been aged for 48 h, and found the cement to show almost no anti-bacterial activity towards *S. auries.* Previous studies have suggested that these cements have some anti-microbial activity against caries-related bacteria such as *Streptococcus mutans* [35] and also the oral bacterium *Candida albicans* [36]. However, this was only when they were very immature. Older specimens showed almost no anti-bacterial behaviour, a feature that was attributed to the change in pH of these cements as they age [36]. Most workers agree that the anti-bacterial character of zinc phosphate cements arises from their early acidity, and that the fully set cement shows no anti-microbial properties. Our results are consistent with these findings.

There have been several studies of novel materials for use in bone-contact applications, with more recent emphasis placed on scaffolds for bone regeneration [16,17,18,19]. These materials typically elicit positive results when tested in cell culture, such as with MG63 cells. Our results for zinc phosphate cement show similarly positive outcomes, with the material promoting cell colonization and proliferation and very high levels of cell viability. Its extract showed no anti-microbial activity. These findings may be considered preliminary in determining the suitability of zinc phosphate cement for use in contact with bone. Further studies are needed to show whether this material provokes any long-term adverse reactions, or degrades with the release of cytotoxic substances. Long-term in vitro results [10] from dentistry suggest that this material is unlikely to have these problems, but further experimental work is necessary to confirm the point.

## 5. Conclusions

This work has demonstrated, for the first time, that MG63 cells are able to colonise the surface of zinc phosphate dental cements and proliferate there. The healthy morphology of the MG63 cells attached to zinc phosphate cement and their continued ability to metabolise indicates that the initial hypothesis being tested, namely that zinc phosphate has acceptable biocompatibility against cultured cells, is correct. Zinc phosphate has been shown to be suitable for further investigation as a material to be used in contact with bone. This could include studies with animal models, such as rabbits or rats. The lack of any adverse biological effects in vitro, in particular the absence of any measurable anti-microbial effects against *S. pyogenes* confirms the high biocompatibility of this material towards cells in general.

## Data Availability

Not applicable.
